# Tetrahedral Framework Nucleic Acid‐Based Delivery of DJ‐1‐saRNA Prevent Retinal Ischaemia–Reperfusion Injury via Inhibiting Ferroptosis

**DOI:** 10.1111/cpr.13820

**Published:** 2025-02-20

**Authors:** Xianggui Zhang, Zhende Deng, Xiaoxiao Xu, Jingyi Zhu, Zhen Huang, Ya Ye, Jingying Liu, Delun Luo, Jinnan Liu, Ming Yan, Yanping Song

**Affiliations:** ^1^ The First School of Clinical Medical Southern Medical University Guangzhou China; ^2^ Innovative Institute of Chinese Medicine and Pharmacy Chengdu University of Traditional Chinese Medicine Chengdu China; ^3^ Department of Ophthalmology General Hospital of Central Theater Command Wuhan China

**Keywords:** DJ‐1 delivery, ferroptosis inhibition, oxidative stress, R28 cells, retinal ischaemic disease, tetrahedral frame nucleic acid

## Abstract

Retinal ischaemia/reperfusion injury (RI/RI) is the primary pathophysiological mechanism underlying retinal ischaemic diseases, potentially resulting in significant and irreversible visual impairment. Currently, there are no effective treatments available for RI/RI, and oxidative stress is a critical factor that contributes to the associated damage. DJ‐1, an important endogenous antioxidant, has been proposed as a promising therapeutic agent for RI/RI owing to its potential for overexpression. In this study, tetrahedral frame nucleic acids (tFNAs) were utilised as an effective delivery vehicle for DJ‐1 small activating RNA (saRNA), resulting in the synthesis of a novel nanocomposite (tFNAs‐DJ‐1‐saRNA). In vitro experiments demonstrated that tFNAs effectively delivered DJ‐1‐saRNA to R28 cells, thus exerting a repair effect on oxidative stress injury. In vivo investigations revealed that the intravitreal injection of tFNAs‐DJ‐1‐saRNA facilitated retinal DJ‐1 gene expression and mitigated retinal atrophy induced by RI/RI. Mechanistically, tFNAs‐DJ‐1‐saRNA activated the xCT/GPX4 pathway, thereby inhibiting ferroptosis, reducing ganglion cell damage and protecting the retinal tissue. In conclusion, this study demonstrated that the tFNAs‐DJ‐1‐saRNA complex can ameliorate RI/RI by inhibiting ferroptosis, suggesting its potential as a novel agent for the treatment of retinal ischaemic diseases.

## Graphical abstract

Illustration of the preparation of tFNAs‐DJ‐1 saRNAs complex and its inhibitory effects on ferroptosis in R28 and retina.

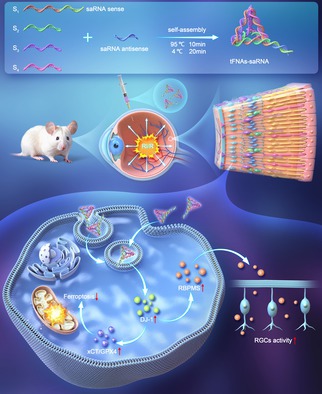

## 1 Introduction

Retinal ischaemic diseases are a series of conditions characterised by retinal ischaemia, including diabetic retinopathy, glaucoma, retinal vascular occlusion and retinopathy of prematurity, all of which can lead to severe and irreversible visual impairment [1]. Retinal ischaemia–reperfusion injury (RI/RI) is a critical pathophysiological process associated with these diseases. Specifically, RI/RI refers to the exacerbation of metabolic imbalances, production of reactive oxygen species (ROS), severe tissue hypoxia and microvascular dysfunction following the restoration of retinal blood perfusion in an ischaemic state [1, 2]. This restoration not only does not recover the original function of the retina but also results in even more severe structural damage, which may be irreversible. Moreover, the reperfusion process significantly amplifies both the innate and adaptive immune responses as well as the activation of cell death pathways [3]. Throughout this process, oxidative stress primarily manifests as free radical damage and glutamate excitotoxicity. This phenomenon can further exacerbate the degeneration of retinal cells, including ganglion and retinal vascular endothelial cells, ultimately leading to irreversible cell damage and death [4, 5, 6]. In addition to apoptosis and necrosis, ferroptosis is a novel mechanism of retinal ganglion cell (RGC) death under RI/RI conditions [7]. Currently, ferroptosis has been confirmed to be closely associated with oxidative stress damage [8]. Consequently, targeting oxidative stress‐induced damage may provide key strategic insights into the development of safe and effective treatment options for RI/RI [4].

DJ‐1 (protein deglucosidase) is a highly conserved 20‐kDa protein encoded by PARK7 that is widely expressed throughout the human body. DJ‐1 functions as both a protease and molecular chaperone, serving as a sensor for cellular oxidative stress. DJ‐1 acts as an endogenous antioxidant through various mechanisms [9], including: (1) regulation of multiple signalling pathways, such as the inhibition of apoptosis signal‐regulated kinase 1 (ASK1)‐induced apoptosis and inhibition of ferroptosis; (2) modulation of other enzymes, molecular chaperones and transcription factors to upregulate the expression of antioxidant genes; and (3) regulation of the thioredoxin and glutathione pathways to mediate antioxidant protection [9]. DJ‐1 plays a role in antioxidative stress damage in various diseases, including diabetes, neurodegenerative disorders, cataract and cardiac, cerebral and renal ischaemia/reperfusion (I/R) injuries [10, 11, 12]. Several studies have investigated the association between DJ‐1 and ferroptosis, revealing that the inhibition of DJ‐1 can increase the sensitivity of tumour cells to ferroptosis inducers [13], whereas the overexpression of DJ‐1 can inhibit ferroptosis in trophoblast cells by activating the Nrf2/GPX4 pathway [14]. DJ‐1 also plays a role in inhibiting ferroptosis in cerebral ischaemic diseases [15]. Therefore, we propose that increasing the expression of DJ‐1 in retinal cells may serve as an effective treatment for RI/RI, as DJ‐1 may inhibit ferroptosis of the retinal ganglion cells by activating the GPX4 pathway, thereby improving the outcomes of RI/RI.

To enhance the in vivo expression level of the DJ‐1 protein and its antioxidant function, previous studies frequently employed viral vectors, including adenoviruses and lentiviruses, to boost DJ‐1 expression [16, 17]. Although these methods have proven effective, they possess inherent drawbacks, such as potential immunogenicity, cytotoxicity and the risk of insertional mutations. Small activated RNA (saRNA) represents a novel gene regulation technology that is capable of inducing the expression of target genes at both the transcriptional and epigenetic levels by targeting gene promoters. This technology has demonstrated significant potential in disease treatment and has been successfully applied in cancer research [18, 19]. Our team has previously shown that saRNA can effectively upregulate the expression of the DJ‐1 gene, a finding that was validated in retinal pigment epithelial (RPE) and vascular endothelial cells [20]. Although saRNA offers advantages such as efficient gene activation, ease of batch synthesis, low toxicity, low cellular uptake rates and susceptibility to nuclease degradation, they pose challenges for effective delivery to target cells in vivo [21]. Consequently, various carriers, including lipids, dendrimers and lipid‐polymer mixtures, are often employed to enhance the pharmacokinetics of saRNA, and the development of new and efficient transport carriers is progressing rapidly [22].

Tetrahedral frame nucleic acids (tFNAs) are novel nanogene and drug delivery systems. They are synthesised through automated hybridisation of four DNA single strands, forming tetrahedral three‐dimensional DNA nanostructures. tFNAs exhibit several advantages, including high penetration, programmability, low immunogenicity, stability in vivo and biological safety, being excellent carriers for molecules, genes and drugs [23, 24, 25]. Furthermore, compared to other transport carriers, tFNAs possess intrinsic antioxidant properties that promote the inhibition of apoptosis and ferroptosis. This capability has been extensively studied and has found initial applications in the treatment of various conditions such as osteoarthritis, acute kidney injury and cardiac I/R injury [23]. In our previous study, we demonstrated that tFNAs effectively reduced the degradation of DJ‐1‐saRNA, highlighting their potential as ideal delivery carriers for DJ‐1‐saRNA. Additionally, tFNAs can mitigate oxidative damage in vascular endothelial and RPE cells [20]. However, the therapeutic efficacy of tFNAs on ganglion cells and RI/RI remains unclear.

In this study, we demonstrated that tFNAs effectively and stably delivered DJ‐1‐saRNA to retinal cells, resulting in a significant increase in DJ‐1 gene expression. Additionally, we used a rat model of RI/RI alongside a cellular model of oxygen–glucose deprivation/reoxygenation (OGD/R) injury to further validate the neuroprotective effects of tFNAs‐DJ‐1‐saRNA, which was shown to inhibit oxidative damage. Mechanistically, tFNAs‐DJ‐1‐saRNA protected RGCs from I/R injury by activating the xCT/GPX4 pathway and inhibiting ferroptosis. This study is the first to explore the combined use of tFNAs and saRNA in the context of RI/RI, offering new insights into the specific mechanisms underlying the action of tFNAs‐DJ‐1‐saRNA in RI/RI treatment. Furthermore, our findings highlight the significant potential of this system for RI/RI applications.

## 2 Materials and Methods

### 2.1 Synthesis and Characterisation of tFNAs‐DJ‐1‐saRNA

The tFNAs‐DJ‐1‐saRNA was synthesised at a concentration of 1 μM using three single‐stranded DNA sequences (S2, S3 and S4; Genscript, Nanjing, China; Table 1), a single‐stranded DNA modified to include a DJ‐1‐saRNA cis‐sense strand (S1‐saRNA sense; Table 1), and one saRNA antisense strand (Table 1). The synthetic method was similar to that of tFNAs. Equal concentrations of the S1‐saRNA sense, saRNA antisense strand, S2, S3 and S4 were combined in TM buffer (50 nM MgCl_2_, 10 nM Tris–HCl, pH = 8.0), mixed thoroughly and then heated to 95°C for 10 min. The mixture was cooled to 4°C for 20 min. The successful synthesis of tFNAs and tFNAs‐DJ‐1‐saRNA was confirmed by polyacrylamide gel electrophoresis (PAGE), high‐performance capillary electrophoresis (HPCE), dynamic light scattering (DLS) and transmission electron microscope (TEM).

**TABLE 1:** Base sequence of each ssDNA and saRNA.

ssDNA	Base sequence (from 5′ to 3′)
S1	TTGACCTGTGAATTATTTATCACCCGCCATAGTAGACGTATCACCAGGCAGTTGAGACGAACATTCCTAAGTCTGAA
S1‐saRNA‐sense	dAdTdTdTdAdTdCdAdCdCdCdGdCdCdAdTdAdGdTdAdGdAdCdGdTdAdTdCdAdCdCdAdGdGdCdAdGdTdTdGdAdGdAdCdGdAdAdCdAdTdTdCdCdTdAdAdGdTdCdTdGdAdAdTdTdTdTdT UGUUCUCUGAGUCCAAGGdTdT
saRNA antisense	CCUUGGACUCAGAGAACATT
S2	ACATGCGAGGGTCCAATACCGACGATTACAGCTTGCTACACGATTCAGACTTAGGAATGTTCG
S3	ACTACTATGGCGGGTGATAAAACGTGTAGCAAGCTGTAATCGACGGGAAGAGCATGCCCATCC
S4	ACGGTATTGGACCCTCGCATGACTCAACTGCCTGGTGATACGAGGATGGGCATGCTCTTCCCG

### 2.2 Cell Culture and Treatment

R28 cells, a rat retinal precursor cell line with differentiation potential, are widely used in the study of retinal neurons and can mimic the physiological and pathological responses of retinal neurons under OGD/R conditions. R28 cells were obtained from ATCC and cultured under OGD/R conditions using Dulbecco's Modified Eagle Medium (DMEM) medium supplemented with 10% Fetal Bovine Serum (FBS) and 1% penicillin and streptomycin (P/S). The cells were maintained at 37°C in an atmosphere containing 95% air and 5% CO_2_ for 24 h. The OGD/R protocol involved culturing the cells in the DMEM medium (95% nitrogen and 5% CO_2_) for 8 h, followed by a medium change to DMEM supplemented with 10% FBS and 1% P/S complete medium, after which the cells were reoxygenated for 24 h at 37°C in an environment of 5% CO_2_ and 95% air. To determine the optimal concentration of tFNAs‐DJ‐1‐saRNA, the cells were co‐cultured with tFNAs‐DJ‐1‐saRNA at concentrations of 125, 250 and 375 nM along with 250 nM tFNA and saRNA. The optimal concentration of tFNAs‐DJ‐1‐saRNA was identified as 250 nM for subsequent studies.

### 2.3 Cellular Uptake

To evaluate the uptake of tFNAs and tFNAs‐DJ‐1‐saRNA by the R28 cells, single‐stranded DNA was fluorescently labelled with Cy5. R28 cells were incubated with Cy5‐tFNAs‐DJ‐1‐saRNA, Cy5‐tFNAs and Cy5‐DJ‐1‐saRNA for 8 h, respectively. Subsequently, the samples were collected and the uptake efficiency was assessed by measuring the fluorescence intensity of Cy5 within the cells using flow cytometry (Attune NxT, Thermo Fisher Scientific, USA).

### 2.4 Cell Viability

Cell viability was assessed using the Cell Counting Kit‐8 (CCK‐8; Yeasen Biotechnology, China), which facilitated the determination of the optimal conditions for modelling and the ideal concentration of tFNAs‐DJ‐1‐saRNA. The cells were treated according to standard cell culture and treatment protocols. Following the instructions provided by CCK‐8, 10 μL of the CCK‐8 reagent was added to each well. The relative cell viability for each group was calculated by absorbance.

### 2.5 Cellular Immunofluorescence Analysis

Cells were plated on dishes and incubated with Cy5‐tFNAs‐DJ‐1‐saRNA, Cy5‐tFNAs or Cy5‐DJ‐1‐saRNA for 16 h. Cells were washed with phosphate‐buffered saline (PBS) and fixed with paraformaldehyde, followed by permeabilisation using Triton X‐100 and incubation with fluorescein isothiocyanate (FITC)‐phalloidine and 4′,6‐diamidino‐2‐phenylindole for cytoskeleton staining and nucleus staining. Finally, the immunofluorescence of the samples was observed using laser scanning confocal microscopy to assess the cellular uptake.

### 2.6 Western Blotting

The complete retinal tissue was carefully separated from the eyeball under a stereoscopic microscope and was subsequently placed into a 1.5‐mL centrifuge tube containing 200 μL of protein extraction buffer (RIPA protein lysis buffer + PMSF protease inhibitor, 1:100). The retinal tissue was homogenised using an ultrasonic fragmentation device until no tissue fragments were visible in the test tube. The centrifuge tubes containing fully lysed retinal tissue proteins were subjected to high‐speed centrifugation at 12000 rpm for 10 min at 4°C. After centrifugation, the supernatant was transferred to a new tube for protein quantification. The extracted proteins were quantified and their concentrations were equilibrated using a BCA Protein Quantification Kit (KeyGEN, Nanjing, China). The supernatant containing 20 μg of protein was heated to 100°C and maintained for 10 min, followed by separation using a 10% SDS‐PAGE gel. The separated proteins were transferred onto a polyvinylidene difluoride (PVDF) membrane (Millipore, Merck, Germany). Subsequently, the PVDF membrane was blocked with a 5% bovine serum albumin (BSA) solution for 1 h at room temperature. The diluted primary antibodies were incubated overnight at 4°C, including PARK7/DJ‐1 (1:1000, ab18257, Abcam), GPX4 (1:5000, ET1706‐45, huabio), xCT (1:1000, HA721868, huabio) and RBPMS (1:1000, abs152188, absin). β‐actin (1:1000, 4970S, CST) served as an internal control. The membrane was incubated with antirabbit IgG and horseradish peroxidase‐conjugated antibody (1:5000, ab6721, Abcam) at ambient temperature for 60 min and visualised using an enhanced chemiluminescence system (ProteinSimple, USA).

### 2.7 Real‐Time Fluorescence Quantitative Polymerase Chain Reaction Analysis

The total RNA was isolated and purified using a FastPure Cell/Tissue total RNA isolation kit (Vazyme Biotechnology, China). Reverse transcription was performed using HiScript III reverse transcriptase (Vazyme Biotechnology, China). All target mRNAs were amplified via quantitative real‐time PCR (qRT‐PCR) using SYBR Green I PCR Master Mix. All BLAST search designs used GAPDH amplification as a control. The corresponding primers are presented in Table 2.

**TABLE 2:** The sequences of PCR primers.

Gene	Primer sequence (5′‐3′)
DJ‐1	Forward: GTGCAGTGTAGCCGTGATGT Reverse: CCTTCACCAAAGCCGACTCA
GAPDH	Forward: TGCACCACCAACTGCTTAG Reverse: GGATGCAGGGATGATGTTC

### 2.8 Lipid ROS

Lipid ROS serves as a reliable marker for detecting ferroptosis in cells. The levels of lipid ROS were quantified using a BODIPY 581/591 C11 fluorescent probe (Thermo Fisher Scientific, USA). Briefly, the cells were incubated with 10 μM BODIPY 581/591 C11 for 30 min at 37°C, followed by three washes with PBS. The level of lipid ROS was assessed by the intensity of red‐green fluorescence observed using fluorescence microscopy, where red fluorescence indicated unoxidised cells and green fluorescence indicated oxidised cells.

### 2.9 Fe^2+^ Detection

The intracellular Fe^2+^ levels were measured using a FerroOrange fluorescent probe (Cell Signalling Technology). The cells were incubated with a working solution of FerroOrange (1 μM) for 30 min at 37°C. Without washing, the intensity of red fluorescence was directly observed using a fluorescence microscope to determine the intracellular Fe^2+^ levels.

### 2.10 Transmission Electron Microscopy

The ultrastructure of the R28 cells in each experimental group was examined using TEM. The cells were collected following standard protocols, prefixed in 3% glutaraldehyde, postfixed in 1% osmium tetroxide, dehydrated using a series of acetone washes and subsequently embedded. The resulting cell‐fixed blocks were sectioned into thin slices measuring 70–90 nm. These sections were then placed on TEM grids and analysed using a TEM JEM‐1400FLASH.

### 2.11 Mitochondrial Membrane Potential

The mitochondrial membrane potential (Δψm) was assessed using JC‐10 mitochondrial staining (Yeasen Biotechnology, China). Briefly, the cells were incubated with JC‐10 (20 μM) for 30 min at 37°C and subsequently washed three times with PBS. Flow cytometry was used for analysis, with R5 representing the population of the cells exhibiting normal mitochondrial membrane potential and R6 representing the population of the cells with decreased or lost mitochondrial membrane potential. The ratio of R5 to R6 in each region was calculated to evaluate the extent of mitochondrial membrane potential damage.

### 2.12 ROS‐Level Detection

Flow cytometry was used to detect the ROS levels in R28 cells subjected to OGD/R. The intracellular ROS levels were assessed using a DCFH‐DA assay kit (Yeasen Biotechnology, China). Following incubation with 10 mM DCFH‐DA in DMEM for 30 min at 37°C, the cells were harvested, washed three times with PBS, suspended in PBS and subsequently analysed using flow cytometry.

### 2.13 Animal Models and Treatments

All experimental procedures were approved by the Ethics Committee of Medical Laboratory Animal Welfare at the Central Theater Command General Hospital (licence number: 2023034). Male Sprague–Dawley (SD) rats (weighing 250–300 g) were obtained from the Animal Experimental Center of the General Hospital of the Central Theater Command. The rats were housed in a controlled environment with sufficient food and water, maintained at a temperature of 22°C ± 1°C and a humidity level of 55% ± 5%. They were subjected to a 12‐h light/dark cycle.

The rats were divided into four groups: control, I/R 1 day group, I/R 3 day group and I/R 7 day group, with three rats in each group (*n* = 3 rats/6 eyes) to assess the retinal damage at different time points following I/R. In subsequent experiments, the rats were categorised into five groups: control, I/R, I/R + saRNA, I/R + tFNAs and I/R + tFNAs‐DJ‐1‐saRNA, with three rats in each group (*n* = 3 rats/6 eyes) to assess the therapeutic effects of different drugs. The therapeutic concentrations of saRNA, tFNAs and tFNAs‐DJ‐1‐saRNA were set at 1 μM, with intravitreal injections administered one day prior to the RI/RI. The RI/RI was induced by the compression of the anterior chamber [26, 27]. The primary procedure involved deeply anaesthetising the rats with a 2% sodium pentobarbital solution (60 mg/kg). A 30G needle was then inserted into the anterior chamber, and the intraocular pressure (IOP) was progressively elevated from 60 to 110 mmHg using an anterior chamber pressor device. This elevated pressure was maintained for 60 min before gradually decreasing back to 60 mmHg.

### 2.14 Retinal Haematoxylin–Eosin Staining and RGCs Counts

All eyeballs were enucleated and the optic nerve was severed with a blade positioned 1 mm posterior to the bulbar wall. The eye cups were fixed, dehydrated and embedded. The eye cup specimens were sectioned into three to four consecutive 4‐μm cryosections through the optic nerve head. Some sections were subsequently baked and stained with haematoxylin—eosin (HE) to facilitate the observation of the histological changes in the retina under a light microscope. RGCs were quantified by examining each section at a high power (10 × 40) across five random, nonrepetitive fields and the average number of RGCs in each field was calculated.

### 2.15 Retinal Immunofluorescence Staining

After incubation with the diluted primary antibody overnight at 4°C, the stained samples were treated with histochemical staining, RBPMS (1:500, ab152188, Absin) and Dapi (1:8, ab104139, Abcam).

### 2.16 Ultra‐Widefield Swept‐Source Optical Coherence Tomography/Optical Coherence Tomography Angiography Imaging

To assess the changes in retinal thickness and blood flow across the different groups, the rat retinas were examined using ultra‐widefield swept‐source optical coherence tomography angiography (UWF‐SS OCTA) (Towardpi Medical Technology Co., China). Following the manufacturer's instructions, the OCTA reference mode was configured as the small‐animal mode. After administering intraperitoneal anaesthesia and performing routine mydriasis, blood flow images covering a 24 × 20 mm^2^ area and B‐scan images depicting various retinal layers were obtained using an imaging system [28, 29].

### 2.17 Statistical Analysis

All data were analysed using GraphPad Prism 9.5 software and presented as mean ± standard deviation (SD). One‐way analysis of variance (ANOVA) was used to compare multiple groups. *p* values less than 0.05 were set as statistically significant.

## 3 Results

### 3.1 Synthesis and Characterisation of tFNAs‐DJ‐1‐saRNA

**FIGURE 1:**
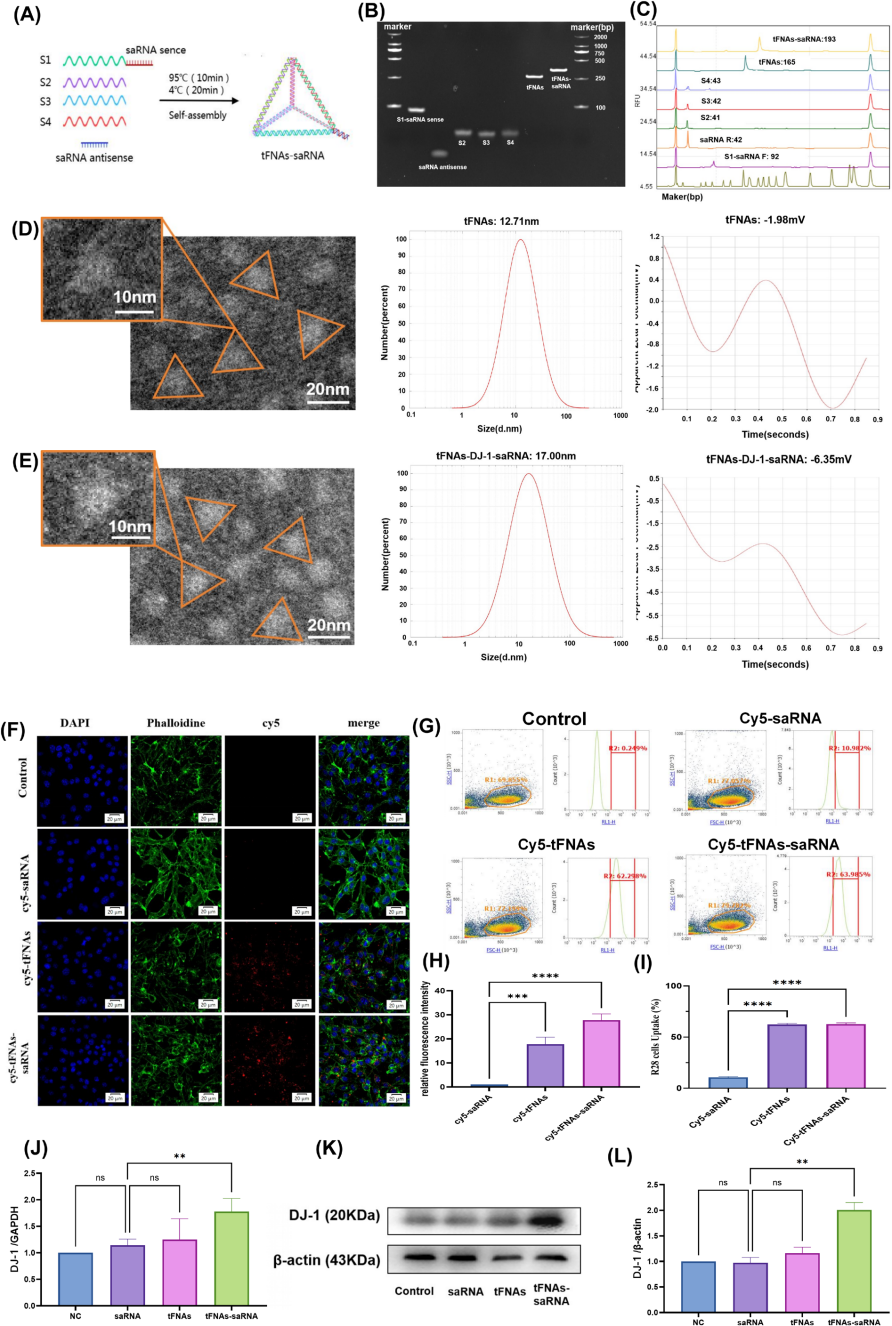
Preparation, characterisation and cellular uptake of tFNAs‐saRNA. (A) Schematic diagram of the preparation of tFNAs‐DJ‐1‐saRNA. (B) Confirmation of the successful synthesis of tFNAs and tFNAs‐DJ‐1‐saRNA by native‐polyacrylamide gel electrophoresis. Lane 1, S1‐saRNA sense; Lane 2, saRNA antisense; Lane 3, S2; Lane 4, S3; Lane 5, S4; Lane 6, tFNAs; Lane 7, tFNAs‐DJ‐1‐saRNA. (C) The successful generation of the tFNAs and tFNAs‐DJ‐1‐saRNA was verified by the high‐performance capillary electrophoresis. (D,E) Transmission electron microscope image of tFNAs and tFNAs‐DJ‐1‐saRNA. Scale bars are 20 nm for transmission electron microscope. Particle size and zeta potential results for the tFNAs and tFNAs‐DJ‐1‐saRNA measured by dynamic light scattering. (F) Cellular uptake of Cy5‐saRNA, Cy5‐tFNAs and Cy5‐tFNAs‐saRNA by R28 for 16 h as determined by immunofluorescence staining (Cy5‐saRNA, Cy5‐tFNAs and Cy5‐tFNAs‐saRNA: Red; nucleus: Blue; cytoskeleton: Green). Scale bars are 20 μm. (G) Quantitative detection and analysis of the uptake rate of Cy5‐saRNA, Cy5‐tFNAs, and Cy5‐tFNAs‐saRNA in R28 cells for 16 h using flow cytometry. (H) Mean fluorescence intensity of Cy5‐tFNAs and Cy5‐tFNAs‐saRNA was significantly higher than that of Cy5‐DJ‐1‐saRNA in R28 cells for 16 h. (I) The uptake rate of Cy5‐tFNAs and Cy5‐tFNAs‐saRNA was significantly higher than that of Cy5‐saRNA in R28 cells for 16 h. (J,K,L) The expression of DJ‐1 mRNA (left) and protein (middle and right) in R28 treated with same concentrations of saRNA, tFNAs and tFNAs‐saRNA for 24 h. Data are presented as mean ± standard deviation (SD) (*n* ≥ 3). Statistical analysis: The ANOVA test was applied; ns, *p* ≥ 0.05; *p < 0.05; **p < 0.01; ***p < 0.001.

Four distinct DJ‐1‐targeted saRNAs were designed (Table 3), and their effects on enhancing DJ‐1 expression in R28 cells were verified by RT‐qPCR. After transfecting the four different saRNAs using a transfection reagent for 24 h, saRNA3 was selected for the synthesis of tFNAs‐DJ‐1‐saRNA, as confirmed by RT‐qPCR (Figure S1). Previous studies demonstrated that tFNAs can readily self‐assemble from four specially designed ssDNA strands (S1, S2, S3 and S4; Table 1) via straightforward annealing. In this study, we modified the DJ‐1‐saRNA sense at the 3′ end of S1, in accordance with prior research, to create a new S1 strand (S1‐saRNA sense, Table 1). The synthesis of tFNAs‐DJ‐1‐saRNA was conducted following the same procedure as that for tFNAs. A schematic of the synthesis of tFNAs‐DJ‐1‐saRNA is shown in Figure 1A.

**TABLE 3:** Base sequence of each saRNA.

saRNA		Base sequence (from 5′ to 3′)
saRNA‐1	Sense	5′‐UAGUAGGAGUUAUGCCAGTT‐3′
	Antisense	5′‐CUGGCAUAACUCCUACUATT‐3′
saRNA‐2	Sense	5′‐UCCAAAACAGUAAGGCUGTT‐3′
	Antisense	5′‐CAGCCUUACUGUUUUGGATT‐3′
saRNA‐3	Sense	5′‐UGUUCUCUGAGUCCAAGGTT‐3′
	Antisense	5′‐CCUUGGACUCAGAGAACATT‐3′
saRNA‐4	Sense	5′‐UCUCUGAGUCCAAGGGAUTT‐3′
	Antisense	5′‐AUCCCUUGGACUCAGAGATT‐3′

8% PAGE was employed to verify the successful synthesis of tFNAs‐DJ‐1‐saRNA. tFNAs‐DJ‐1‐saRNA (lane 7) exhibited the slowest migration compared to S1‐saRNA sense (lane 1), saRNA antisense (lane 2), ssDNAs (lanes 3–5) and tFNAs (lane 6) (Figure 1B). HPCE revealed that the size of tFNAs‐DJ‐1‐saRNA was 193 bp, confirming its successful synthesis (Figure 1C). The TEM images demonstrated that both tFNAs and tFNAs‐DJ‐1‐saRNA were triangular in the 2D plane (Figure 1D,E), which is consistent with the results of previous studies [20, 26]. Additionally, particle size and zeta potential measurements obtained through DLS indicated that the particle size of tFNAs was approximately 12.71 nm, whereas that of tFNAs‐DJ‐1‐saRNA was approximately 17.00 nm (Figure 1D,E). The zeta potential of tFNAs‐DJ‐1‐saRNA (approximately −6.35 mV) was lower than that of tFNAs (approximately −1.98 mV) (Figure 1D,E). These results suggest that DJ‐1‐saRNA was successfully loaded onto tFNAs.

### 3.2 Cellular Uptake of tFNAs‐DJ‐1‐saRNA and DJ‐1 Expression

To investigate the potential protective effect of tFNAs‐DJ‐1‐saRNA on RI/RI, R28 cells were used for in vitro cellular experiments. We initially assessed the intracellular uptake of tFNAs‐DJ‐1‐saRNA using immunofluorescence and flow cytometry. Confocal laser scanning microscopy revealed that the majority of the cells incubated with Cy5‐tFNAs‐DJ‐1‐saRNA and Cy5‐tFNAs exhibited red fluorescence, whereas no significant red fluorescence was detected with Cy5‐saRNA (Figure 1F,H). The flow cytometry results indicated that over 60% of the cells were able to uptake Cy5‐tFNAs‐DJ‐1‐saRNA or Cy5‐tFNAs after 12 h of incubation (Figure 1G,I). In contrast, the rate of uptake of Cy5‐saRNA was relatively low, approximately 10%. These findings suggest that tFNAs can enhance the uptake of DJ‐1‐saRNA in R28 cells.

Subsequently, we employed RT‐PCR and WB analyses to assess DJ‐1 expression following incubation with tFNAs‐DJ‐1‐saRNA (Figure 1J–L), revealing that both mRNA and the protein levels of DJ‐1 in R28 cells were significantly elevated after treatment with 125 nM tFNAs‐DJ‐1‐saRNA. In contrast, DJ‐1‐saRNA did not significantly affect DJ‐1 expression. Therefore, tFNAs can be utilised as an effective delivery system for DJ‐1‐saRNA, facilitating the upregulation of the target gene expression and promoting the associated biological effects.

### 3.3 Antioxidant Effect of tFNAs‐DJ‐1‐saRNA

To investigate the antioxidant effect of tFNAs‐DJ‐1‐saRNA, R28 cells were subjected to various OGD/R conditions (OGD 2, 4, 6, 8, 10, 12 and 24 h; R 24 h). The CCK‐8 assay indicated that cell viability gradually decreased with OGD duration. Under OGD 8 h/R 24 h conditions, the cell viability decreased to approximately 60% (Figure S2). The effects of the different concentrations of tFNAs‐DJ‐1‐saRNA on the R28 cells were also examined, revealing that tFNAs‐DJ‐1‐saRNA at concentrations ranging from 0 to 500 nM (0, 62.5, 125, 250 and 500 nM) did not significantly affect the cell viability (Figure S3). In subsequent experiments, the R28 cells were cultured under OGD 8 h/R 24 h conditions with the addition of tFNAs‐DJ‐1‐saRNA to evaluate their potential to enhance cell viability. tFNAs‐DJ‐1‐saRNA (125, 250 and 375 nM) significantly improved the viability of the R28 cells, with 250 nM tFNA‐DJ‐1‐saRNA exhibiting the most pronounced effect. In contrast, DJ‐1‐saRNA alone did not affect the viability of the R28 cells (Figure S4).

**FIGURE 2:**
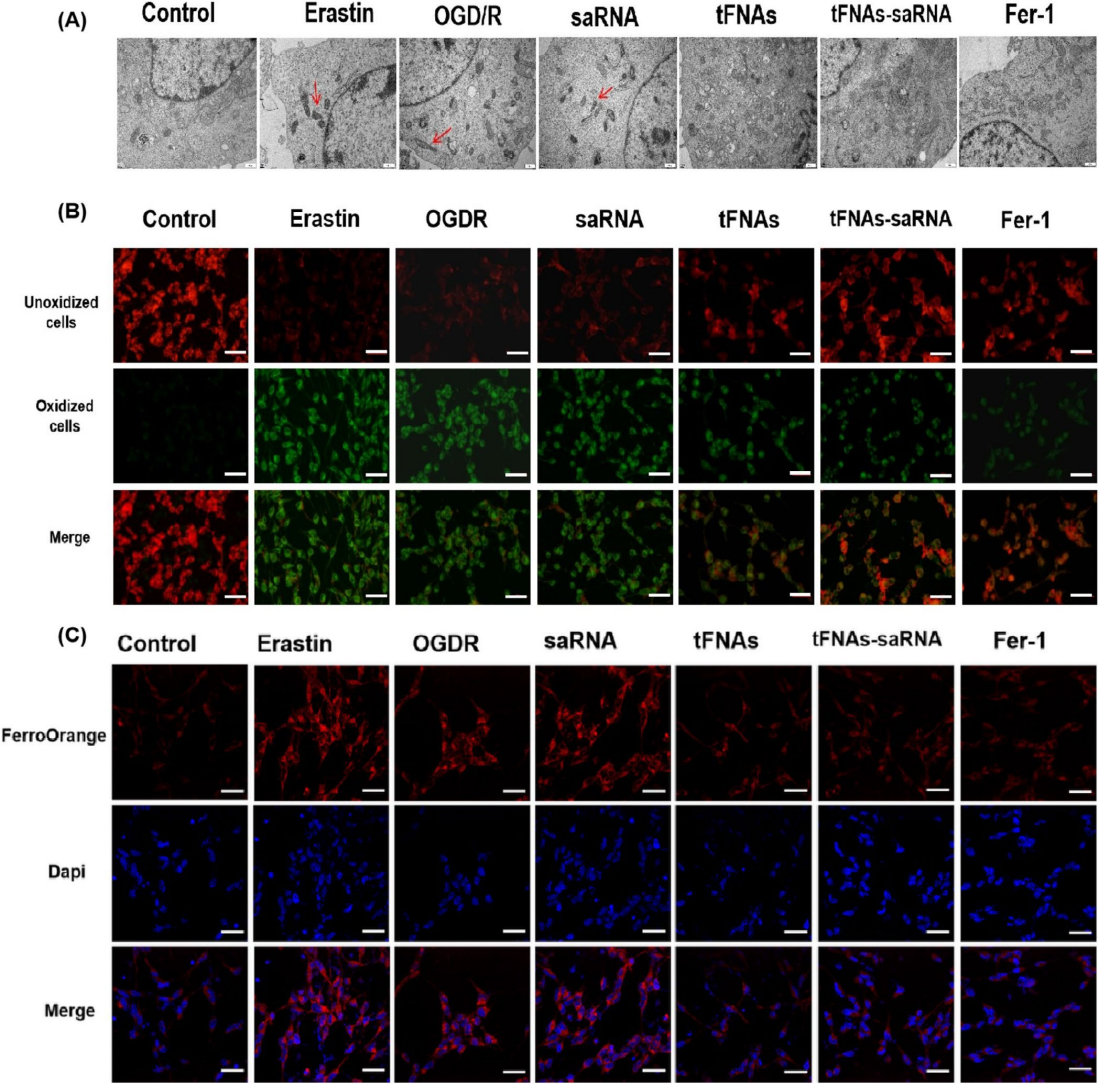
tFNAs‐saRNA inhibited ferroptosis in R28 cells. (A) Transmission electron microscope images of the overall and mitochondrial structure in R28 after exposure to erastin, OGD/R, OGD/R + saRNA, OGD/R + tFNAs, OGD/R + tFNAs‐saRNA, OGD/R + Fer‐1. Ferroptosis‐like changes in mitochondrial morphology (red arrows). Scale bar = 500 nm (B) Intracellular lipid ROS detected by BODIPY 581/591 C11 dye. The cells from each group were stained with BODIPY 581/591 C11 reagent and then analysed by immunofluorescence. Scale bar = 50 μm (C) The intracellular Fe^2+^ levels in each group was detected by immunofluorescence. Scale bar = 50 μm.

These results aligned with the antioxidant effects of tFNAs and tFNAs‐DJ‐1‐saRNA reported previously [20, 26]. Ferroptosis is characterised by oxidative damage. To determine whether ferroptosis occurs in R28 cells under OGD/R conditions, we measured the ROS levels and assessed ferroptosis following treatment with various drugs (saRNA, tFNAs and tFNAs‐DJ‐1‐saRNA) at a concentration of 250 nM. A detailed examination of the ultrastructural changes in the cells was conducted via TEM, revealing cytoplasmic and organelle swelling, as well as mitochondrial atrophy in R28 cells subjected to OGD/R injury (Figure 2A). The alterations in mitochondria within the OGD/R group were particularly pronounced, exhibiting smaller and darker mitochondria, destruction of mitochondrial cristae and disruption of the mitochondrial outer membrane. These changes are similar to those observed in the mitochondria of the ferroptosis agonist‐treated (erastin) group. All these morphological changes are indicators of ferroptosis. Treatment with tFNAs‐DJ‐1‐saRNA significantly mitigated the pathological changes in R28 cells, indicating an improvement in mitochondrial morphology comparable to that observed in the ferroptosis inhibitor‐treated (Fer‐1) group (Figure 2A).

Lipid peroxidation serves as the gold standard for detecting ferroptosis via the measurement of the intensity of red‐green fluorescence using fluorescence microscopy. Red fluorescence indicates unoxidised cells and green fluorescence indicates oxidised cells. We found that green fluorescence was significantly increased and red fluorescence was significantly decreased in OGD/R, saRNA and erastin groups compared with the control group. Conversely, after treatment with tFNAs, tFNAs‐DJ‐1‐saRNA and Fer‐1, we observed an increase in red fluorescence and a decrease in green fluorescence (Figure 2B). Intracellular Fe^2+^ is a typical marker of ferroptosis. Therefore, the intracellular Fe^2+^ levels were further investigated. The Fe^2+^ levels (red fluorescence) were significantly elevated in the OGD/R and saRNA groups after OGD/R injury. However, after treatment with tFNAs, tFNAs‐DJ‐1‐saRNA and Fer‐1, the Fe^2+^ levels (red fluorescence) were significantly reduced (Figure 2C). Notably, tFNAs and tFNAs‐DJ‐1‐saRNA significantly inhibited OGD/R‐induced ferroptosis.

**FIGURE 3:**
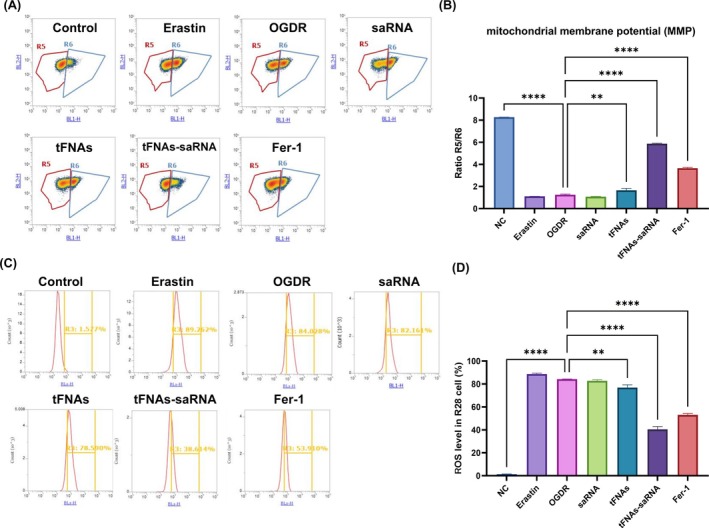
tFNAs‐saRNA improved mitochondrial membrane potential and ROS levels. (A,B) Detection of mitochondrial membrane potential with JC‐10 dye. Each group of cells was stained with JC‐10 reagent, and then analysed by flow cytometry. (C,D) Quantitative detection and analysis of the intracellular ROS levels after different treatment regimes in R28 using flow cytometry. Data are presented as means ± SD from three independent experiments. Data are presented as mean ± standard deviation (SD) (*n* ≥ 3). Statistical analysis: The ANOVA test was applied; ns, *p* ≥ 0.05; *p < 0.05; **p < 0.01; ***p < 0.001.

Given the significant changes in mitochondrial morphology observed in the R28 cells following OGD/R injury, we further evaluated the alterations in mitochondrial function. Mitochondria are crucial organelles responsible for ATP production, redox signalling and cell death. The JC‐10 probe was employed to assess the mitochondrial membrane potential. Flow cytometric analysis revealed that the ratio of healthy to damaged mitochondria (R5/R6) significantly decreased after OGD/R injury. However, this ratio notably improved following tFNAs‐DJ‐1‐saRNA treatment (R5/R6). Furthermore, tFNAs‐DJ‐1‐saRNA effectively mitigated OGD/R‐induced cell stress and cytotoxicity (Figure 3A,B).

The ROS levels were assessed using flow cytometry, revealing a significant increase in R28 cells following OGD/R injury. Moreover, treatment with tFNAs‐DJ‐1‐saRNA effectively reduced the ROS levels in the R28 cells (Figure 3C,D).

### 3.4 In Vivo Therapeutic Effect of tFNAs‐DJ‐1‐saRNA

**FIGURE 4:**
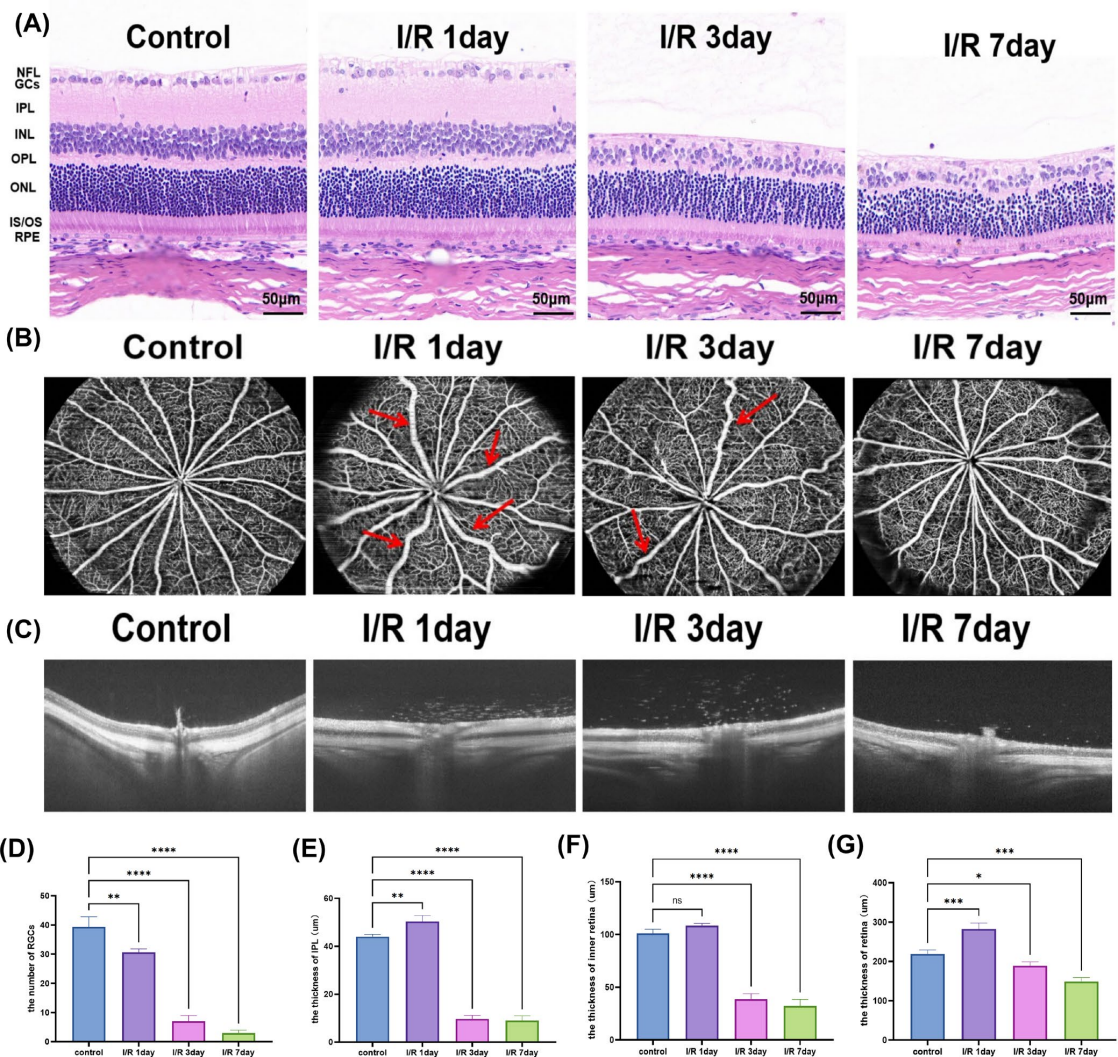
Retinal thickness and vascular changes after retinal ischaemia–reperfusion injury. (A) HE staining images at 1, 3 and 7 days after retinal ischaemia–reperfusion injury. Scale bar = 50 μm. (B) OCTA images at different times after retinal ischaemia–reperfusion injury. The large retinal vein was tortuous and dilated after RI/RI (red arrow). (C) B‐scan images of retina at different time points after retinal ischaemia–reperfusion injury were observed by OCT. (D–F) The number of RGCs, the thickness of IPL layer and the thickness of inner retina were analysed by HE staining. (G) Quantitative analysis of total retinal thickness in different groups by OCT. Data are presented as means ± SD from three independent experiments. Data are presented as mean ± standard deviation (SD) (*n* ≥ 3). Statistical analysis: The ANOVA test was applied; ns, *p* ≥ 0.05; *p < 0.05; **p < 0.01; ***p < 0.001.

Section 3.3 presented the protective effects of tFNAs‐DJ‐1‐saRNA on OGD/R‐injured R28 cells in vitro. To further investigate the changes in the retina following RI/RI and the therapeutic effect of tFNAs‐DJ‐1‐saRNA in vivo, we established a rat model of RI/RI by inducing high IOP through anterior chamber perfusion. We employed HE staining and optical coherence tomography (OCT) to assess the retinal thickness 1, 3 and 7 days post‐RI/RI, alongside OCTA to evaluate alterations in the retinal blood vessels. HE staining revealed that after 1 d of I/R, the thickness of IPL increased and the number of RGCs decreased. At 3 and 7 d postmodelling, the thickness of the inner retina decreased, the number of RGCs continued to decline, IPL became thinner and the inner nuclear layer exhibited disorganisation (Figure 4A,D–F). The OCT findings corroborated these results, showing increased retinal thickness, oedema in the inner retina and ischaemic changes in the outer retina 1 d after I/R. Notably, atrophy of the inner retina and a progressive decrease in the total retinal thickness were observed 3 and 7 d postmodelling (Figure 4C,G). The OCTA results indicated that the large retinal vein was tortuous and dilated, with an enlarged vein diameter 1 d after I/R. The tortuosity of the retinal vein was alleviated by day 3 postmodelling. On day 7, the diameter of the venous lumen gradually returned to normal levels (Figure 4B).

**FIGURE 5:**
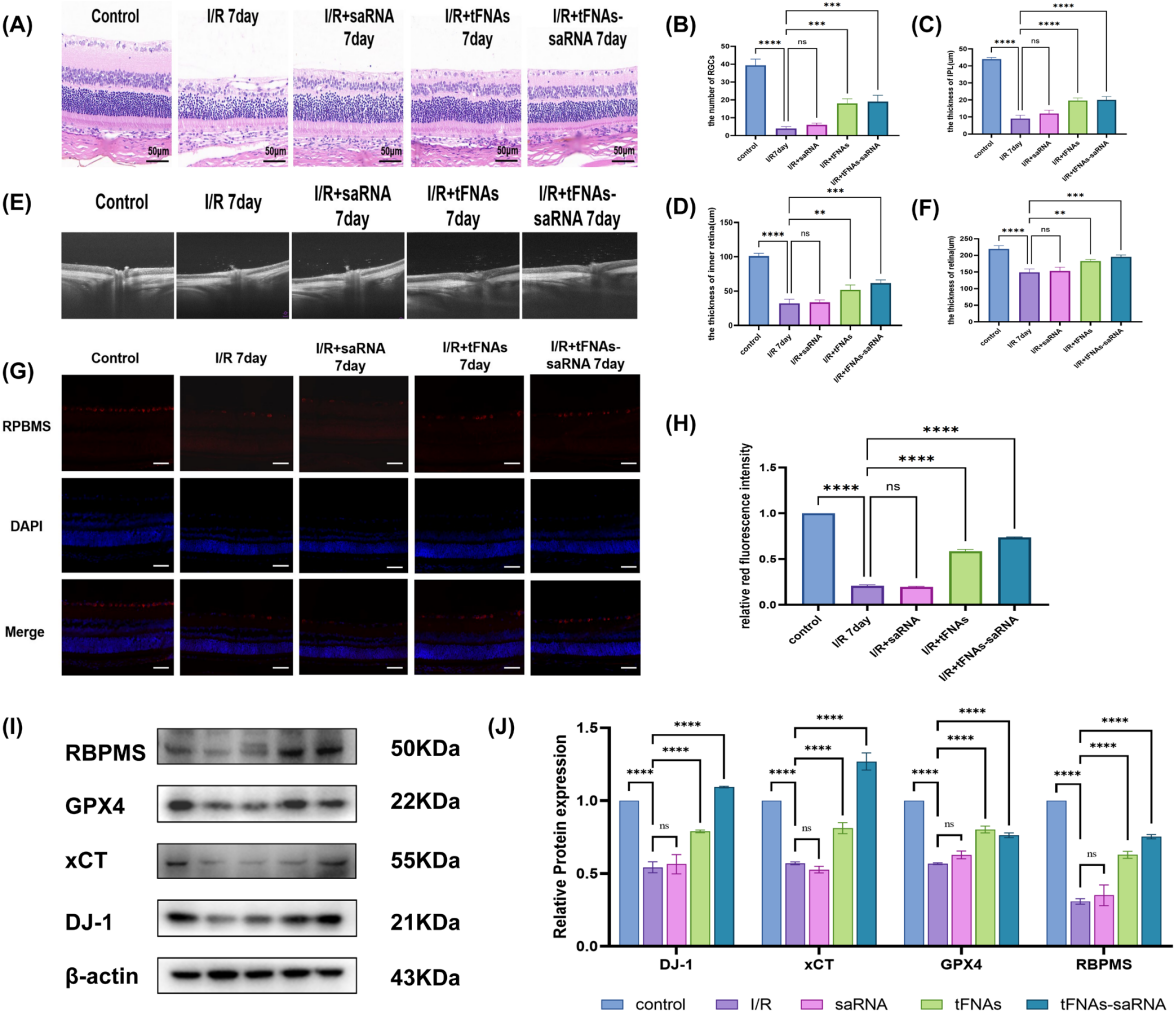
tFNAs‐saRNA protects against retinal injury in rats. (A) HE staining images 7 days after intravitreal injection of saRNA, tFNAs and tFNAs‐saRNA intervention in retinal ischaemia–reperfusion injury. Scale bar = 50 μm. (B–D) The number of RGCs, the thickness of IPL layer and the thickness of the inner retina were analysed by HE staining. (E) OCT images of retinal ischaemia–reperfusion injury 7 days after intervention with different drugs. (F) Quantitative analysis of total retinal thickness in different groups by OCT. (G) Immunofluorescence staining of RBPMS (retinal ganglion cell active protein, red fluorescence) after 7 days of retinal ischaemia–reperfusion injury treated with different drugs. (H) Quantitative analysis of the fluorescence intensity of RBPMS in different groups by immunofluorescence. (I,J) Western blot was used to detect the expression of DJ‐1 protein, ferroptosis‐related proteins (xCT, GPX4) and RBPMS proteins in different groups. Data are presented as mean ± standard deviation (SD) (*n* ≥ 3). Statistical analysis: The ANOVA test was applied; ns, *p* ≥ 0.05; *p < 0.05; **p < 0.01; ***p < 0.001.

Due to the dilution of the vitreous cavity itself and the presence of metabolic enzymes within the tissue, a higher concentration of intravitreal drugs is necessary to enhance drug stability and efficacy in vivo. Previous studies reported a drug concentration of 1000 nM^26^. SaRNA, tFNAs and tFNAs‐DJ‐1‐saRNA were administered into the vitreous cavity prior to modelling, and the effects of these drugs were evaluated 7 d postmodelling. HE staining revealed a significant reduction in the number of RGCs as well as in the thickness of IPL and the inner retina 7 d after model establishment. Treatment with tFNAs and tFNAs‐DJ‐1‐saRNA ameliorated the loss of RGCs and mitigated atrophy of IPL and the inner retina (Figure 5A–D). OCT was employed to further assess the retinal condition of the rats in vivo, revealing a significant reduction in the total retinal thickness and observable retinal atrophy 7 d after modelling. Although treatment with saRNA alone did not yield significant changes, administration of tFNAs and tFNAs‐DJ‐1‐saRNA effectively improved this pathological state (Figure 5E,F).

RBPMS is a critical protein in neuronal survival, synapse formation and repair, and serves as the active protein in RGCs. Therefore, RBPMS expression provides insights into the activity of RGCs [30, 31]. Immunofluorescence staining revealed that the expression of the RBPMS protein (indicated by red fluorescence) in the ganglion cell layer following RI/RI was significantly lower than that observed in the normal group. Furthermore, RBPMS protein expression in the saRNA group was comparable to that in the RI/RI group, whereas the expression levels increased following treatment with tFNAs and tFNAs‐DJ‐1‐saRNA. These findings suggest that drug treatment enhanced the activity of RGCs and improved their function (Figure 5G,H).

To further investigate the effect of tFNAs‐DJ‐1‐saRNA on DJ‐1 protein expression in the context of RI/RI and explore its potential therapeutic mechanisms, we conducted WB experiments. A significant decrease in DJ‐1 protein expression was observed after RI/RI. However, the DJ‐1 protein levels were elevated following treatment with tFNA and tFNAs‐DJ‐1‐saRNA, with the tFNAs‐DJ‐1‐saRNA group exhibiting higher expression than the tFNA group. Additionally, we observed a significant reduction in RBPMS expression after RI/RI, which was restored following tFNA and tFNAs‐DJ‐1‐saRNA administration, corroborating the findings of the immunofluorescence experiments. Furthermore, we found that tFNAs‐DJ‐1‐saRNA enhanced the expression of the ferroptosis‐protective proteins GPX4 and xCT in the retinal tissue, thereby mitigating ferroptosis‐related damage. This suggests that tFNAs‐DJ‐1‐saRNA may safeguard the retinal tissue by inhibiting ferroptosis, ultimately reducing ganglion cell damage during RI/RI (Figure 5I,J).

## 4 Discussion

RI/RI is common in various fundus diseases, including diabetic retinopathy, glaucoma, retinal vascular occlusion and retinopathy of prematurity. RI/RI is an important pathophysiological process in retinal ischaemic diseases, in which oxidative stress damage plays an important role. Recently, oxidative stress‐induced ferroptosis has garnered increasing attention as a novel mechanism of cell death associated with RI/RI [7, 32]. Currently, the primary clinical treatments for retinal ischaemic diseases include intravitreal injections of antivascular endothelial growth factor (anti‐VEGF) drugs, glucocorticoids and laser therapy. However, the therapeutic effects of these approaches are limited. Consequently, exploring new pharmacological agents for the treatment of RI/RI based on their underlying pathological mechanisms is of paramount importance.

In our previous study, we successfully synthesised tFNAs‐DJ‐1‐saRNA and utilised tFNAs as efficient transport carriers for delivery to cells [20]. To the best of our knowledge, this is the first study to investigate the regulatory effects of tFNAs‐DJ‐1‐saRNA on RI/RI. In vitro experiments demonstrated that tFNAs‐DJ‐1‐saRNA significantly increased the protein levels of DJ‐1 in R28 cells. Following OGD/R‐induced damage in R28 cells, treatment with tFNAs‐DJ‐1‐saRNA markedly enhanced cell survival and reduced ROS levels. Additionally, tFNAs‐DJ‐1‐saRNA treatment significantly decreased the lipid ROS and Fe^2+^ levels while improving the mitochondrial morphology and membrane potential in of the damaged R28 cells, thereby inhibiting ferroptosis. In addition, we revealed that tFNAs possess excellent carrier functions as well as antioxidant properties. Previous studies have demonstrated that tFNAs positively regulate various mammalian cell functions, including the promotion of cell migration, proliferation and differentiation, while exhibiting anti‐inflammatory, antioxidant, anti‐infection and immunomodulatory effects [33, 34, 35]. Furthermore, tFNAs significantly reduce the apoptosis of RGCs induced by oxidative stress and protect RGCs by modulating the expression of oxidative stress‐related enzymes and apoptosis‐related proteins [26, 36]. Although our findings align with those of previous studies, we observed that tFNAs‐DJ‐1‐saRNA exhibited superior therapeutic effects compared to tFNAs. Our results further substantiate the notion that DJ‐1 functions as an antioxidant and protects cells from oxidative stress‐induced damage by inhibiting ferroptosis.

Animal experiments were conducted to further verify the protective effects of tFNAs‐DJ‐1‐saRNA against RI/RI. Our findings indicated that retinas subjected to RI/RI exhibited temporal gradient changes at different time points. On the first day postmodelling, retinas displayed signs of ischaemic oedema due to acute injury. However, by the third and seventh days, the retinal oedema resolved, revealing progressive atrophic changes. Furthermore, OCTA was used for the first time to assess the retinal vascular injury in rats subjected to RI/RI. On the first day after modelling, an increase in the tortuosity and dilation of the large retinal vein was observed, along with a reduction in retinal ischaemia and vascular density. These findings suggest that the retinal tissue response is sustained after I/R injury and is closely linked to the production of various oxidative and inflammatory factors in the retina, which subsequently contribute to vascular damage and cell death [37, 38].

Following previous studies [26, 39], we administered an intravitreal injection of tFNAs‐DJ‐1‐saRNA to treat RI/RI. Our results demonstrated that tFNAs‐DJ‐1‐saRNA effectively mitigated retinal atrophy and ganglion cell loss by day 7 postmodelling. To further investigate the specific mechanism of action of tFNAs‐DJ‐1‐saRNA in the treatment of RI/RI, we evaluated the expression levels of the ferroptosis‐related proteins xCT and GPX4 in retinal tissue to assess the occurrence of ferroptosis. Our results indicated that the levels of the ferroptosis‐protective proteins xCT and GPX4 were significantly reduced after modelling but increased after treatment with tFNAs‐DJ‐1‐saRNA. Such a finding further confirms that tFNAs‐DJ‐1‐saRNA mitigates ganglion cell damage by inhibiting ferroptosis in RI/RI, thereby protecting the optic nerve tissue. Additionally, we demonstrated that tFNAs could exert antioxidant functions by inhibiting ferroptosis in RI/RI, which is consistent with a previous study [40]. In an acute kidney injury model, tFNAs inhibited the cleavage of poly (ADP‐ribose) polymerase, thereby reducing the apoptosis of renal tubular epithelial cells, downregulating GPX4 expression and ROS production and inhibiting ferroptosis [40]. This further substantiates the antioxidant role of tFNAs in inhibiting ferroptosis in various diseases. However, tFNAs‐DJ‐1‐saRNA showed a more significant effect than tFNAs.

Due to experimental limitations, our study has several constraints. First, the OCTA system used lacks the capability to quantitatively assess retinal vascular tortuosity, so this parameter was not included. Second, retinal vascular changes following pharmacological treatment were not visualised using OCTA. Furthermore, visual function tests such as VEP, ERG and EOG were not performed. Future studies will address these limitations by optimising the experimental design and incorporating more comprehensive evaluations of retinal vasculature and optic nerve function.

## 5 Conclusion

In summary, the observation that the tFNAs‐DJ‐1‐saRNA complex can enhance RI/RI by inhibiting ferroptosis provides a theoretical basis and foundation for the future application of tFNAs‐DJ‐1‐saRNA in clinical practice. It is anticipated that tFNAs‐DJ‐1‐saRNA will emerge as an effective treatment for retinal ischaemic diseases.

## Author Contributions

Xianggui Zhang and Zhende Deng contributed equally to this work. Study conception and design: Yanping Song, Ming Yan, Zhen Huang, Ya Ye, Delun Luo. Acquisition of data: Xianggui Zhang, Zhende Deng, Xiaoxiao Xu, Jingying Liu. Analysis and interpretation of data: Xianggui Zhang, Zhende Deng, Jingyi Zhu, Jinnan Liu.

## Conflicts of Interest

The authors declare no conflicts of interest.

## Supporting information


**Figure S1.** The expression of DJ‐1 in R28 cells was detected by real‐time quantitative PCR. Real‐time quantitative PCR (RT‐qPCR) was used to measure the expression of DJ‐1 mRNA in R28 cells at 24 h after transfection with 4 different saRNAs. GAPDH was used as an internal control. Statistical analysis: the ANOVA test was applied; ns, *p* ≥ 0.05, *, *p* < 0.05; **, *p* < 0.01; ***; *p* < 0.001; ****p < 0.0001.

**Figure S2.** Cell viability of R28 cells cultured under different oxygen–glucose deprivation/reoxygenation (OGD/R) conditions. R28 cells were cultured under different OGD/R conditions (OGD2, 4, 6, 8, 10, 12 and 24 h, R24 h), and the cell viability was detected by CCK‐8. Cell viability of R28 treated with different OGD/R conditions was time‐dependent. Compared with the control group, the cell viability of R28 was significantly decreased after 4–24 h OGD/R24h treatment (all *p* < 0.01). The cell viability of OGD8 h/R24 h was about 60%, which was the best condition for establishing the model. Statistical analysis: the ANOVA test was applied; ns, *p* ≥ 0.05, *, *p* < 0.05; **, *p* < 0.01; ***; *p* < 0.001, ****p < 0.0001.

**Figure S3.** Cell viability of R28 cells cultured with different concentrations of tFNAs‐saRNA. After R28 cells were co‐cultured with different concentrations of tFNAs‐saRNA (62.5/125/250/500 nM) for 24 h, the cell viability of R28 cells was detected by CCK‐8. Compared with the control group, the viability of R28 cells cultured with different concentrations of tFNAs‐saRNA for 24 h did not change significantly (all *p* ≥ 0.05). Statistical analysis: the ANOVA test was applied; ns, *p* ≥ 0.05, *, *p* < 0.05; **, *p* < 0.01; ***; *p* < 0.001, ****p < 0.0001.

**Figure S4.** Screening for optimal therapeutic concentration of tFNAs‐saRNA. The morphology of R28 cells was observed by optical microscope, and the cell viability of R28 cells was detected by CCK‐8 assay. Light microscopy showed that after OGDR injury, the cell density was reduced and the cell morphology was swollen and degenerated. There was no significant improvement in cell morphology in the saRNA co‐culture group. The cell density and morphology were improved in the tFNAs and different concentrations of tFNAs‐saRNA co‐culture groups. CCK‐8 assay showed that tFNAs and different concentrations of tFNAs‐saRNA could significantly improve the cell viability of R28 cells (all *p* < 0.05), and the 250 nM tFNAs‐saRNA had the best effect. Statistical analysis: the ANOVA test was applied; ns, *p* ≥ 0.05, *, *p* < 0.05; **, *p* < 0.01; ***; *p* < 0.001, ****p < 0.0001.

## Data Availability

The data that support the findings of this study are available on request from the corresponding author.
