# Supplementary figures and images for: Tetrahedral Framework Nucleic Acid‐Based Delivery of DJ‐1‐saRNA Prevent Retinal Ischaemia–Reperfusion Injury via Inhibiting Ferroptosis
Source: Cell Prolif. 2025 Feb 20;58(7):e13820. doi: 10.1111/cpr.13820 (PMC12240634; doi:10.1111/cpr.13820)

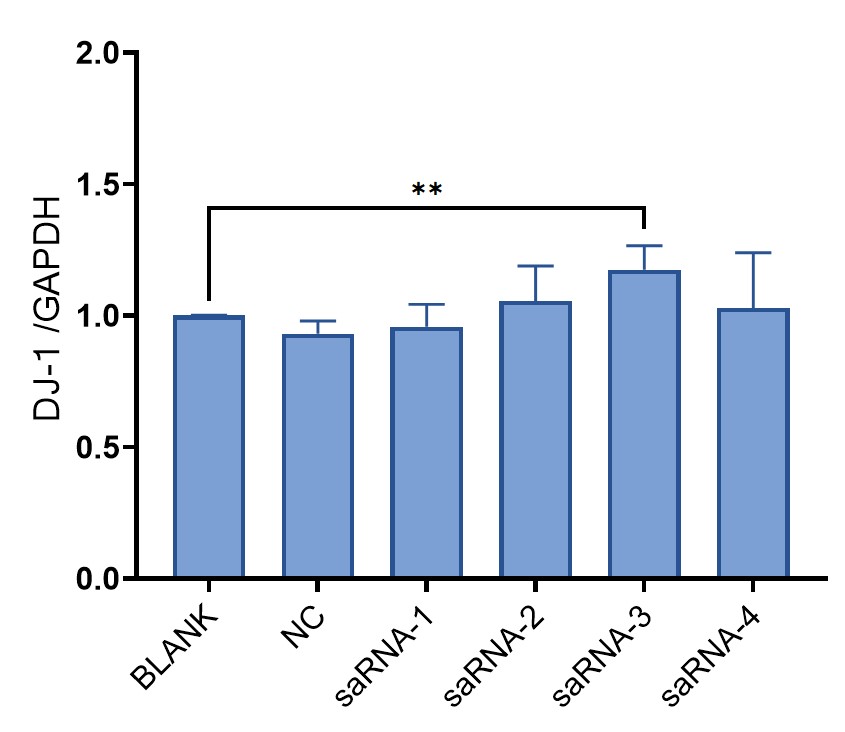

Supplement: Supplementary file 1 — Figure S1. The expression of DJ‐1 in R28 cells was detected by real‐time quantitative PCR. Real‐time quantitative PCR (RT‐qPCR) was used to measure the expression of DJ‐1 mRNA in R28 cells at 24 h after transfection with 4 different saRNAs. GAPDH was used as an internal control. Statistical analysis: the ANOVA test was applied; ns, p ≥ 0.05, *, p < 0.05; **, p < 0.01; ***; p < 0.001; ****p < 0.0001. [file CPR-58-e13820-s003.jpg]

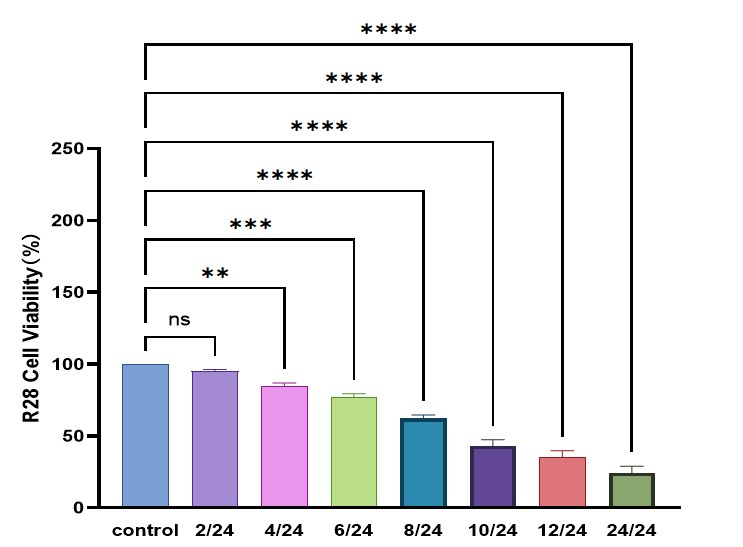

Supplement: Supplementary file 2 — Figure S2. Cell viability of R28 cells cultured under different oxygen–glucose deprivation/reoxygenation (OGD/R) conditions. R28 cells were cultured under different OGD/R conditions (OGD2, 4, 6, 8, 10, 12 and 24 h, R24 h), and the cell viability was detected by CCK‐8. Cell viability of R28 treated with different OGD/R conditions was time‐dependent. Compared with the control group, the cell viability of R28 was significantly decreased after 4–24 h OGD/R24h treatment (all p < 0.01). The cell viability of OGD8 h/R24 h was about 60%, which was the best condition for establishing the model. Statistical analysis: the ANOVA test was applied; ns, p ≥ 0.05, *, p < 0.05; **, p < 0.01; ***; p < 0.001, ****p < 0.0001. [file CPR-58-e13820-s002.jpg]

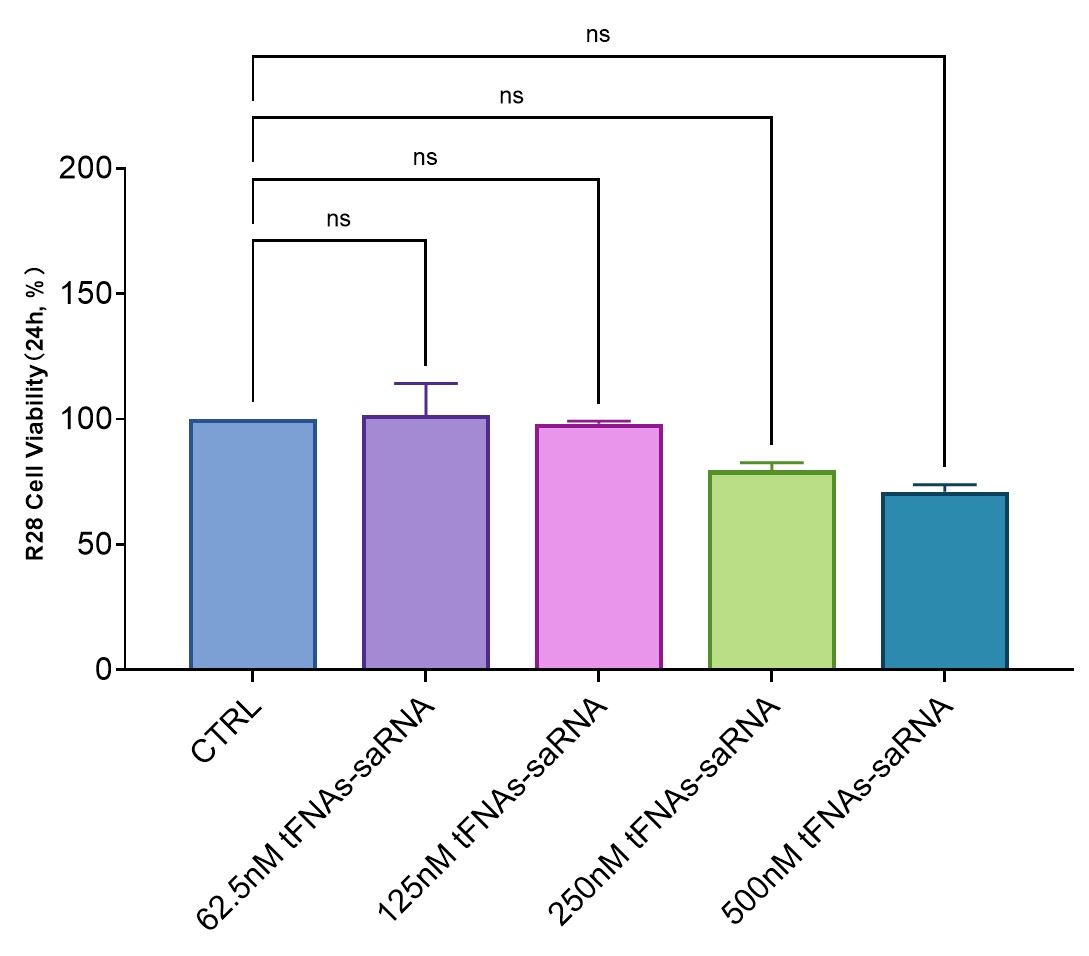

Supplement: Supplementary file 3 — Figure S3. Cell viability of R28 cells cultured with different concentrations of tFNAs‐saRNA. After R28 cells were co‐cultured with different concentrations of tFNAs‐saRNA (62.5/125/250/500 nM) for 24 h, the cell viability of R28 cells was detected by CCK‐8. Compared with the control group, the viability of R28 cells cultured with different concentrations of tFNAs‐saRNA for 24 h did not change significantly (all p ≥ 0.05). Statistical analysis: the ANOVA test was applied; ns, p ≥ 0.05, *, p < 0.05; **, p < 0.01; ***; p < 0.001, ****p < 0.0001. [file CPR-58-e13820-s004.jpg]

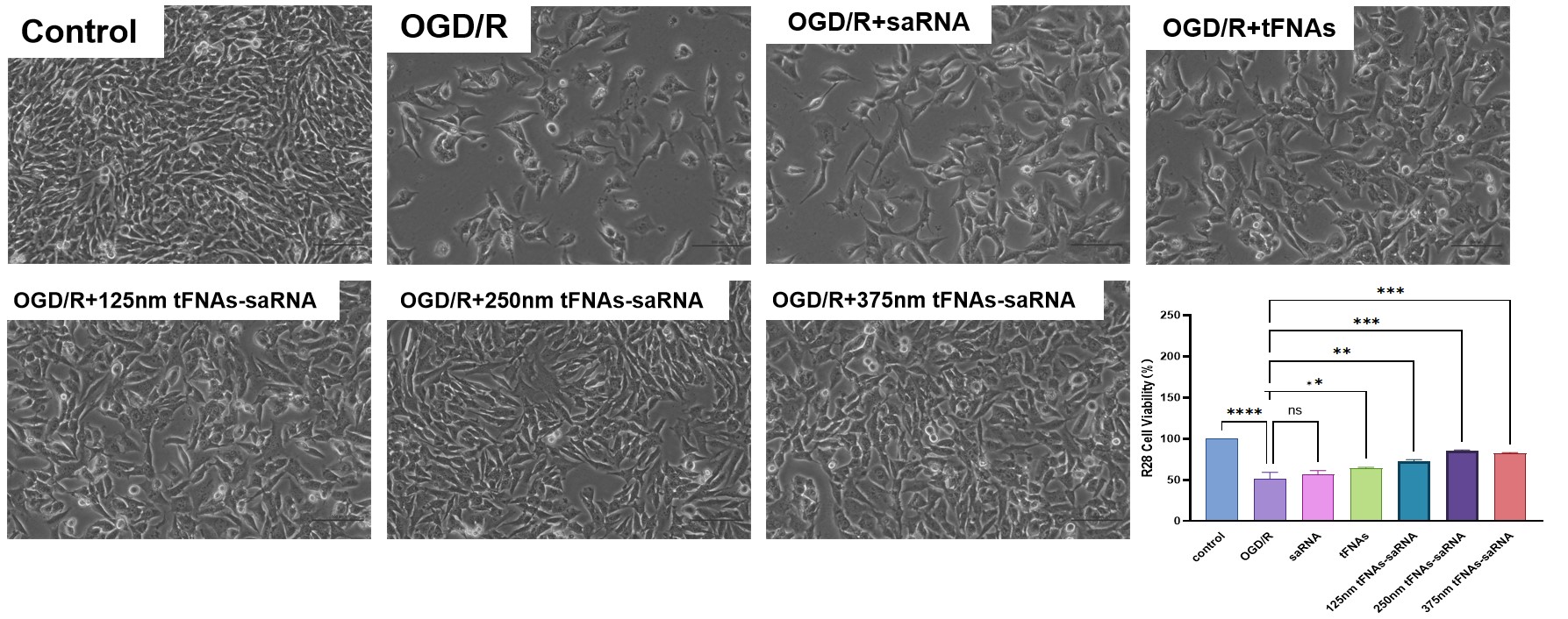

Supplement: Supplementary file 4 — Figure S4. Screening for optimal therapeutic concentration of tFNAs‐saRNA. The morphology of R28 cells was observed by optical microscope, and the cell viability of R28 cells was detected by CCK‐8 assay. Light microscopy showed that after OGDR injury, the cell density was reduced and the cell morphology was swollen and degenerated. There was no significant improvement in cell morphology in the saRNA co‐culture group. The cell density and morphology were improved in the tFNAs and different concentrations of tFNAs‐saRNA co‐culture groups. CCK‐8 assay showed that tFNAs and different concentrations of tFNAs‐saRNA could significantly improve the cell viability of R28 cells (all p < 0.05), and the 250 nM tFNAs‐saRNA had the best effect. Statistical analysis: the ANOVA test was applied; ns, p ≥ 0.05, *, p < 0.05; **, p < 0.01; ***; p < 0.001, ****p < 0.0001. [file CPR-58-e13820-s001.jpg]
